# Morphology, membrane nanostructure and stiffness for quality assessment of packed red blood cells

**DOI:** 10.1038/s41598-017-08255-9

**Published:** 2017-08-10

**Authors:** E. Kozlova, A. Chernysh, V. Moroz, V. Sergunova, O. Gudkova, E. Manchenko

**Affiliations:** 1 Federal Research and Clinical Center of Intensive Care Medicine and Rehabilitology, V.A. Negovsky Scientific Research Institute of General Reanimatology, Moscow, Russian Federation; 2 Federal State Autonomous Educational Institution of Higher Education I.M. Sechenov First Moscow StateMedical University of the Ministry of Health of the Russian Federation, Moscow, Russian Federation

## Abstract

Transfusion of packed red blood cells (PRBC) to patients in critical states is often accompanied by post-transfusion complications. This may be related with disturbance of properties of PRBC and their membranes during long-term storage in the hemopreservative solution. *The purpose of our work* is the study of transformation of morphology, membranes stiffness and nanostructure for assessment of PRBC quality, *in vitro*. By atomic force microscopy we studied the transformation of cell morphology, the appearance of topological nanodefects of membranes and by atomic force spectroscopy studied the change of membrane stiffness during 40 days of storage of PRBC. It was shown that there is a transition period (20–26 days), in which we observed an increase in the Young’s modulus of the membranes 1.6–2 times and transition of cells into irreversible forms. This process was preceded by the appearance of topological nanodefects of membranes. These parameters can be used for quality assessment of PRBC and for improvement of transfusion rules.

## Introduction

A large number of post-transfusion complications causes intensive discussion in the scientific community about quality assessment of packed red blood cells (PRBC) with different storage time^1–5^. In accordance with the recommendations of WHO^6^ PRBC for transfusion can be stored at 4 °C during 42 days. In a number of European countries, this period is limited to 35 days^7^. The criteria for the suitability of PRBC for its use in clinical practice is the level of hemolysis (not more than 0.8%), percentage erythrocytes that is removed within a 24 hours after transfusion (no more than 25%) and recovery of parameters *in vivo* during 24 hours^8–10^. If the first criterion is possible to be measured *in vitro*, the second should be determined *in vivo*, that may be is very problematic. Therefore, *in vivo* studies do not provide an opportunity to make unambiguous conclusions about the suitability of PRBC^11, 12^. Although the hemolysis level can correspond the above requirements, *in vitro* studies show that biochemical and metabolic indicators significantly worsen already in the first two weeks and especially in the second half of the PRBC storage period^4, 10, 13–17^. Morphology of PRBC is disturbed^18–21^, and reversibility of cells forms is inversely proportional to storage time^9, 22^.

Quality of PRBC is largely determined by the shape of cells and the structure of their membranes^12^. Since the RBC membrane itself is the main target for pathological influence, it is the main determinant of the clinical effects of PRBC storage^23^. Changes in the forms of erythrocytes and disturbances in the structure of their membranes can lead to decrease of deformability of PRBC, to worsening of rheological properties of blood, to weakening of gas transport function^24^ and, ultimately, to a decrease of PRBC quality. The development and justification of additional quality criteria for PRBC and their suitability for transfusion is one of the key problems in the medicine of critical states.

The effective methods for studying of morphology of erythrocytes, nanostructure and stiffness of their membranes are atomic force microscopy (AFM) and the atomic force spectroscopy (AFS)^25–29^. These methods do not require preliminary modification of the object, and the resolution limit of atomic force microscopes is less than one nanometer, which makes it possible to study in detail structure of cell membranes. Also it is possible to measure Young’s modulus of native cells. Atomic force microscopy has advantages over optical microscopy and electron microscopy.

The purpose of the work is the study of transformation of morphology, membranes stiffness and nanostructure for assessment of PRBC quality, *in vitro*.

## Results

**Figure 1:**
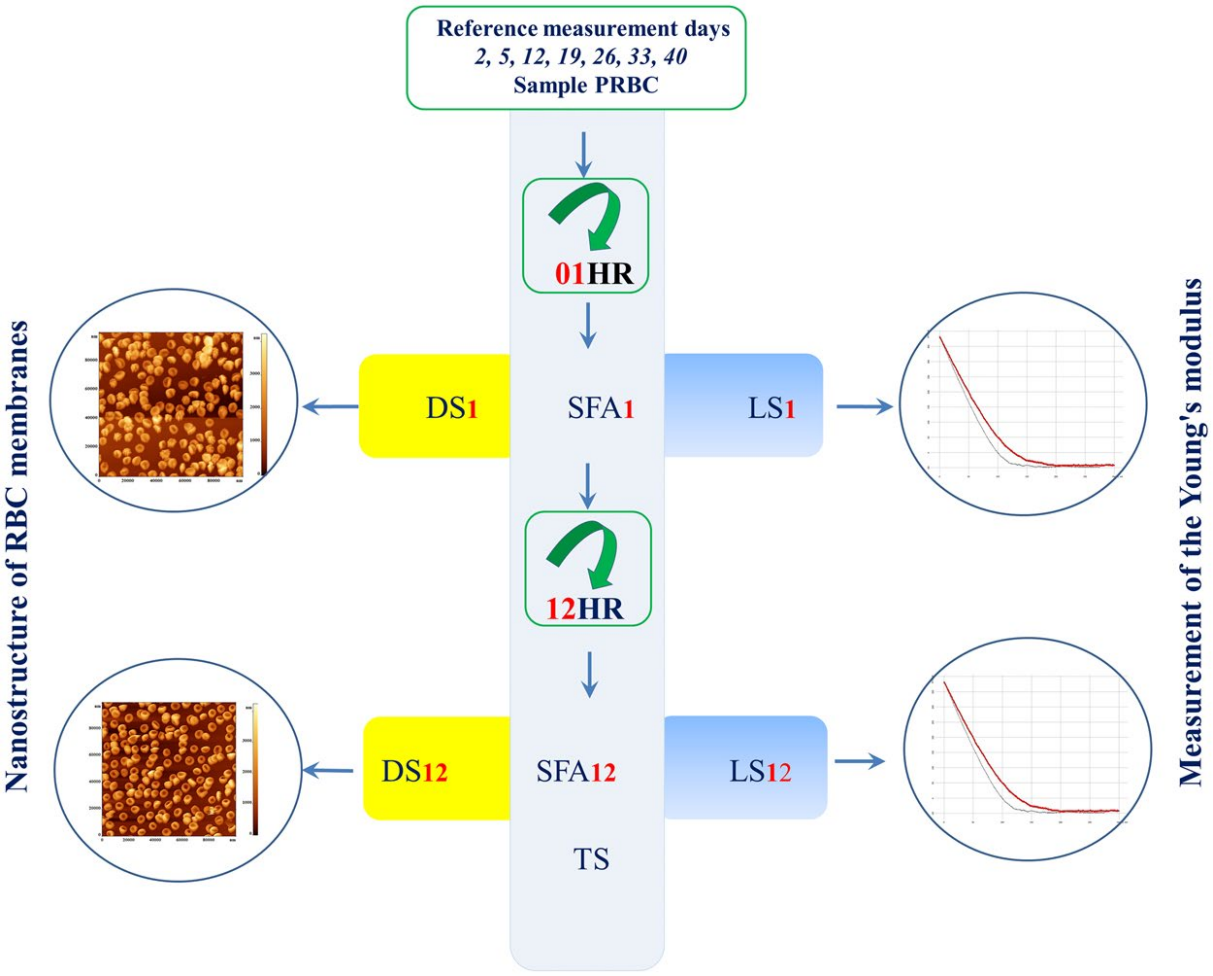
Design of experiments. DS – Dry sample, LS – Liquid sample, SFA – Sample For Analysis, TS - Thermostat, 01 HR - 1 hour of rotation, 12 HR - 12 hours of rotation. Index 1–1 hour of rotation, index 12–12 hours of rotation.

The measurements were carried out at the “control”, and also at some “intermediate” storage days according to the scheme shown in Fig. 1. Samples were taken from bag with PRBC into tubes and were set in a rotator, placed in a thermostat with a temperature 37 °C. After reaching of PRBC sample the set temperature (during 1 hour), 0.5 volume from each tube was taken for carrying out measurements. The rest sample was stored in the thermostat on the rotator else for 12 hours. This allowed to check whether the parameters of the cells remain the same, improve or worsen. Dry monolayers (DS) were made to obtain images of cells and membranes nanostructure by AFM; monolayers of native cells in a liquid medium (LS) were used for measuring of membrane stiffness.

This scheme of experiments was carried out for each of 8 bags. According to this scheme, during the entire experiment 56 experiments were conducted for control days and 12 for additional days.

### Young’s modulus (stiffness) of RBC membranes

Young’s modulus (*E*) is one of the main indicators of mechanical state of blood cells membranes, in particular their stiffness. During the storage *E* changes and consequently, the mechanical properties of cells also change. The values of Young’s modules of membranes of different cells even on one monolayer can differ substantially. Below the statistical distributions of *E* for different days of PRBC storage and conditions are presented.

**Figure 2:**
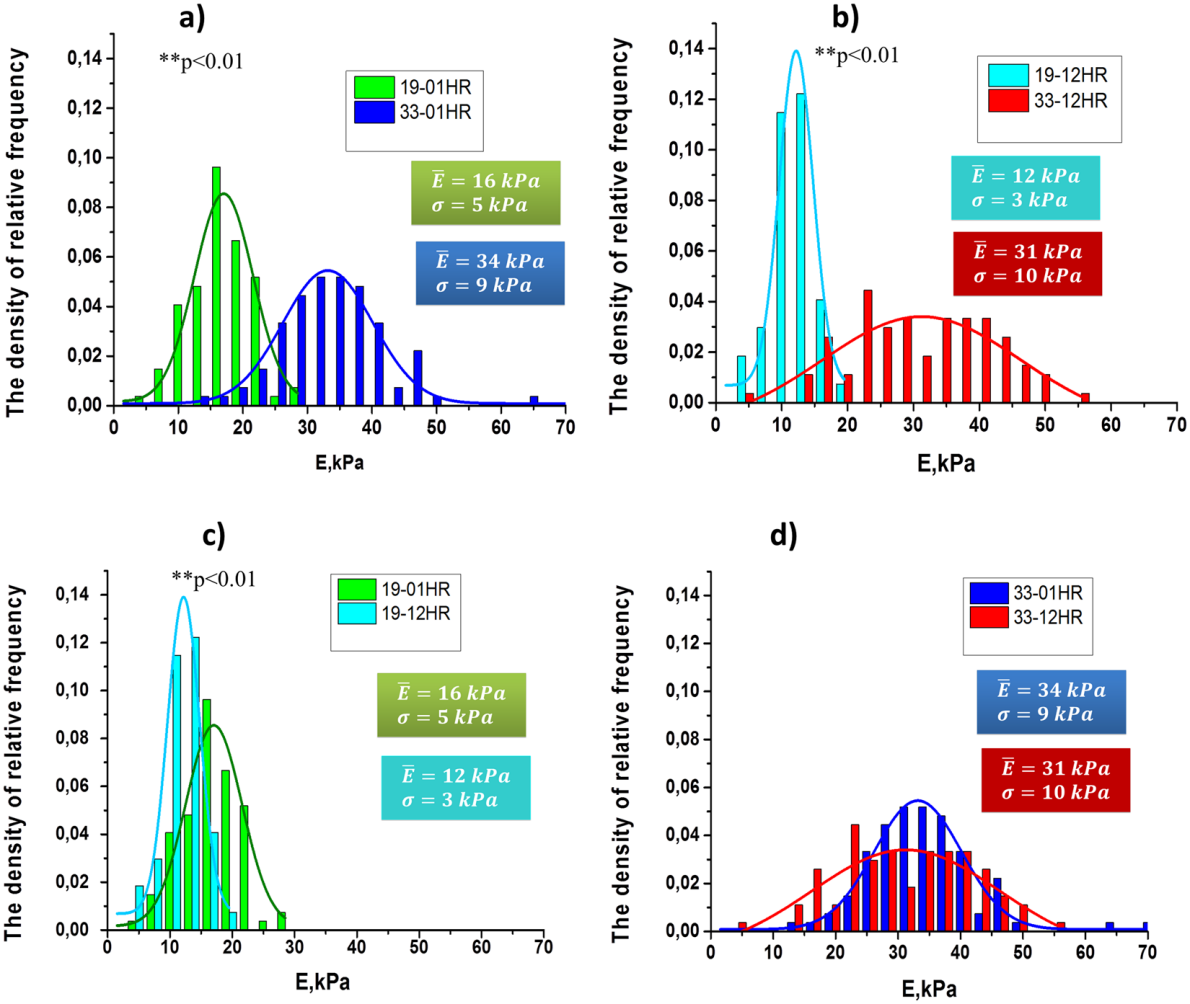
Histograms of the density of relative-frequency for values E for 19^th^ and 33^th^ days of PRBC storage and their approximation by the normal Gaussian distribution law. The density of relative frequencies is plotted along the ordinate axis, and the values of *E* is plotted along the abscissa axis. (**a**) 19–01 HR – 19^th^ storage day, after 1 hour of rotation, 33–01 HR - 33^th^ storage day, after 1 hour of rotation (^**^p < 0.01, differences are statistically significant). (**b**) 19–12 HR- 19^th^ storage day, after 12 hour of rotation, 33–12 HR-33 days of storage, after 12 hours rotation (^**^p < 0.01, differences are statistically significant). (**c**) 19–01 HR and 19–12 HR - 19 days of storage, after 01 hour and 12 hours of rotation respectively (^**^p < 0.01, differences are statistically significant).(**d**) 33–01 HR and 33–12 HR - 33 days of storage, after 01 hour and 12 hours of rotation respectively (differences are not statistically significant). E_m_ – the mean value of Young’s modulus, σ - the standard deviation.

One of the measurement tasks was to compare the values *E* for the first half (initial) period of storage (IPS) and the second half (end) period of storage (EPS). For IPS the cells up to 20–21 days of storage are considered, for EPS - after 21–23 days and until the end of storage period. In the first half of storage, on the 5^th^, 12^th^ and 19^th^ days, the value of *E* varied within 16 ± 5 kPa. After 19 and to 40 storage days, the value of *E* increased significantly. For 01 HR average *E* increased to 34 kPa on the 33^d^ day of storage, that is almost 2 times, and *σ* rose from 5 to 9 kPa (Fig. 2a).

After 12 hours of rotation, the tendency was the same. On the 19^th^ day of storage *E* = 12 ± 3 kPa, and on the 33^d^ day mean *E* increased to the value 31kPa (Fig. 2b). That is, the value *E* for EPS increased by 2.6 times compared to IPS. Also the longer the PRBC was stored, the greater was dispersion of *E*. So, on the 19^th^ day σ = 3 kPa, and by the 33^th^ day it increased to σ = 10 kPa.

The data of comparison of Young’s modulus distributions for 19 and 33 days of storage for rotation during 1 hour and 12 hours are shown in Fig. 2c,d.

For the 19 days of storage (Fig. 2c), the initial (01 HR) values of the average Young’s modulus were *E* = 16 ± 5 kPa. During 12 hours of rotation RBC became more elastic, their Young’s modulus decreased: *E* = 12 ± 3 kPa. The differences between *E* for 01 HR and 12 HR were statistically significant (^**^p < 0.01).

For cells of the second half (EPS) of storage for 01 HR modulus *E* = 34 ± 9 kPa, and after 12 hours of rotation it was *E* = 31 ± 10 kPa. Thus the Young’s modulus for EPS cells substantially exceeding the parameters of the first half of storage and remained practically unchanged for 98% RBC after 12 hours of rotation (Fig. 2d).

**Figure 3:**
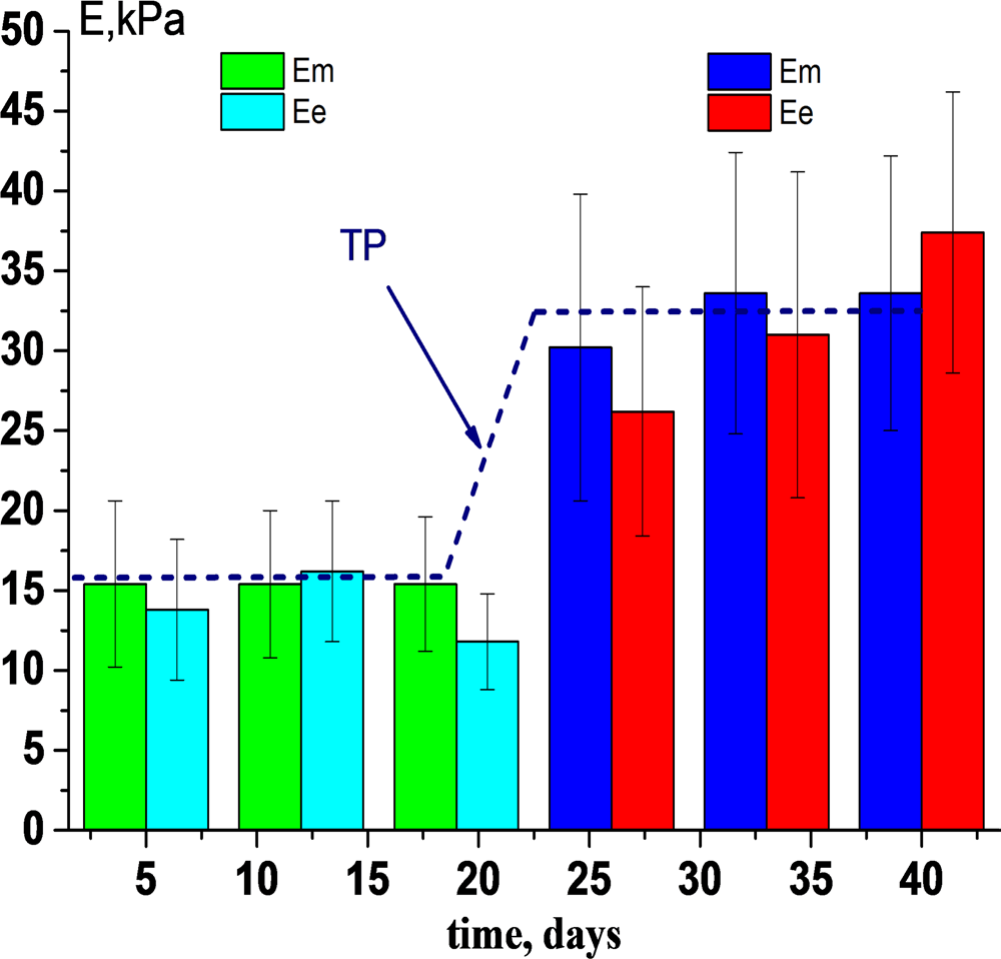
Change in the value of Young’s modulus (*Е*) during the PRBC storage. 01HR (E_m_) and 12HR (E_e_) - 1 and 12 hours of rotation at 37 °С, respectively. IPS - period before 20 day; EPS - the period after 26 and up to 40 days; TP - transition period. ^**^p < 0.01, differences between *E* for IPS and EPS were statistically significant.

The transition of relatively soft membranes (14–16) kPa - (IPS cells) to rigid (31–34) kPa began to develop after the 19–21 day of storage of PRBC - the transition period. In just 7 days the Young’s modulus of RBC membranes increased more than two times (Fig. 3).

IPS cells after 12 hours of rotation became softer (Young’s modulus decreased), but in EPS cells this could be differently. For example, on the 40^th^ day of storage, the Young’s modulus of the RBC increased after the rotation of 12 hours (Fig. 3). The stiffness of EPS cells did not return to the values of the IPS cells.

- In this section we showed that there is a transition period (20–26 days) during long-term storage of PRBC, in which there is a sharp and irreversible increase in the Young’s modulus of membranes 1.6–2 times.

### RBC morphology

Another important indicator cells state is the morphology of PRBC. We studied the morphology of cells throughout the entire period of storage of PRBC.

**Figure 4:**
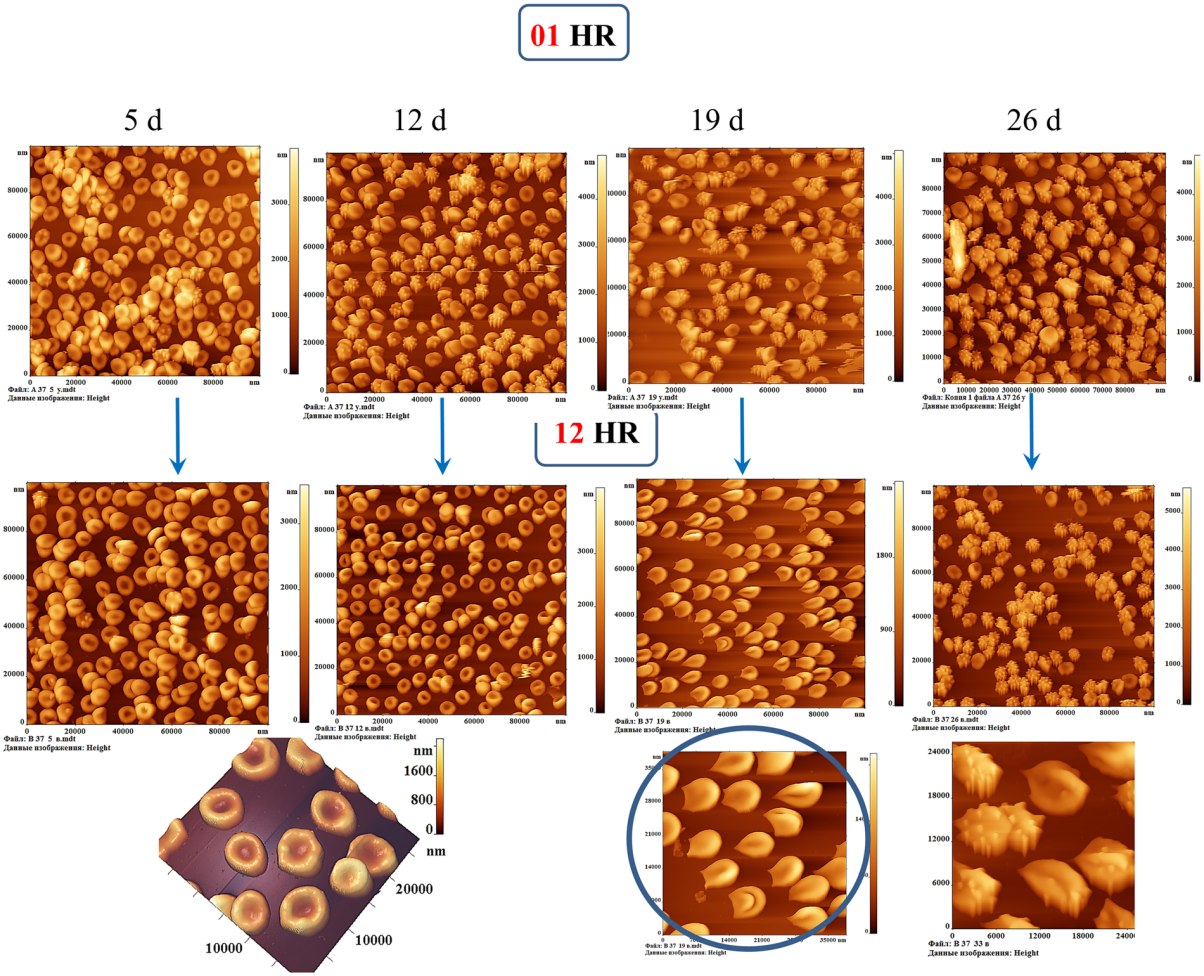
Images of PRBC in the AFM field on the 5^th^, 12^th^, 19^th^ and 26^th^ days of storage of PRBC. The first row - 1 hour (01HR), the second - 12 hours (12HR) of rotation of PRBC in thermostat at the temperature of 37 °C. Scan size is 100 × 100 μm^2^. In the below row the cells after 12 hours of rotation are presented on an enlarged scale. The teardrop cells (dacrocytes) (lemon-like cells) and keratocytes are highlighted by blue circle.

AFM images of cells for different control storage days for 1 hour and 12 hours of PRBC rotation are shown in Fig. 4. For the indicated days, these changes can be seen by comparing the corresponding images along the vertical (Fig. 4, shown by arrows).

**Figure 5:**
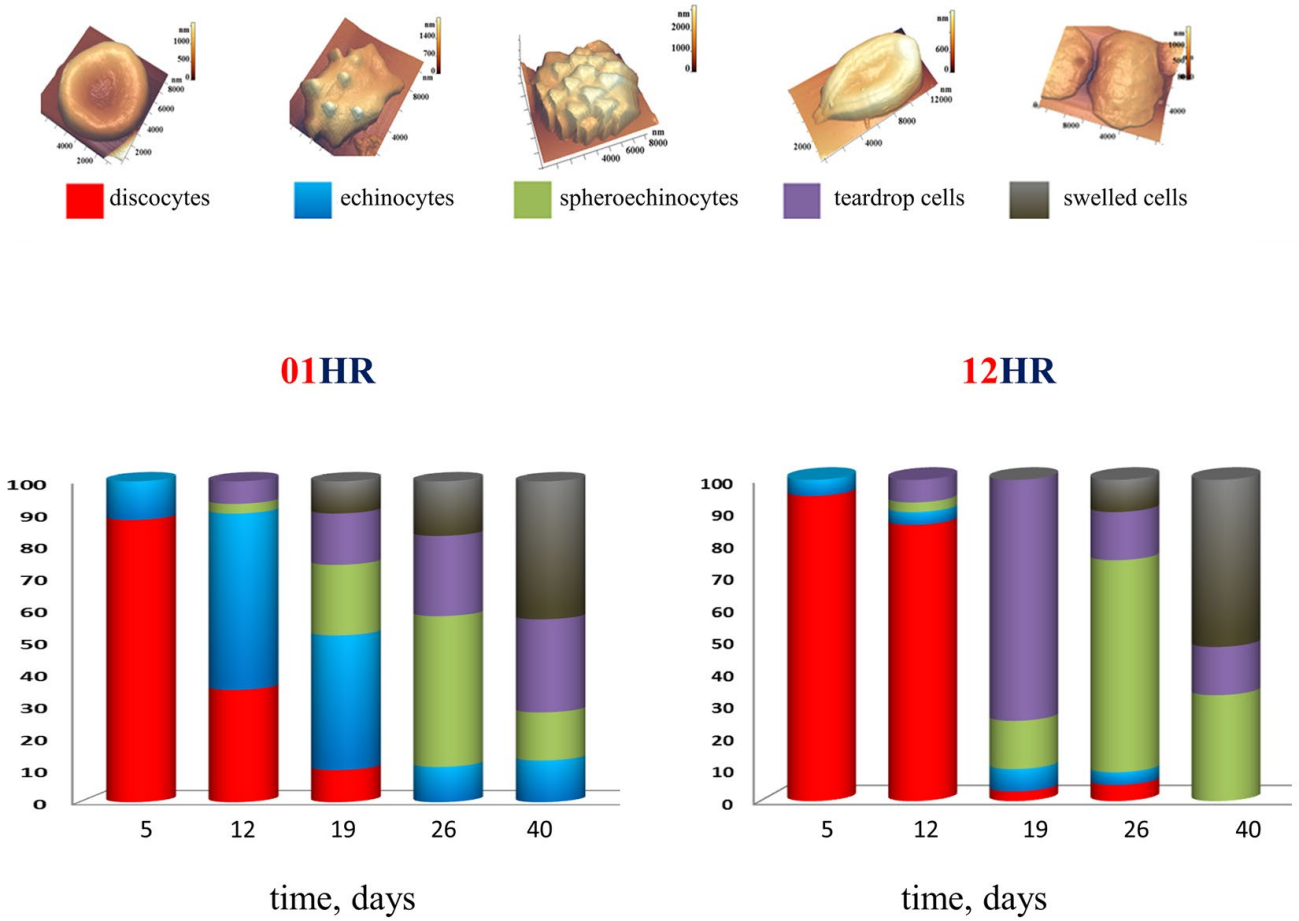
Distribution of the basic forms of PRBC during PRBC storage for 1 hour (01HR) and 12 hours (12HR) of rotation. In our experiments there were indicated: discocytes, echinocytes, spheroechinocyte, teardrop (dacrocyte) (lemon-like), swelled cells (in forms spheres and ovals).

On the 5^th^ day of storage of PRBC discocytes (88 ± 15)% and echinocytes (12 ± 4)% were presented on a monolayer (Figs 4 and 5d).

On the 12^th^ day in monolayer there were presented dyscocytes 35 ± 4%, echinocytes 55 ± 5%, spheroechinocytes 3 ± 1%, teardrop cells 7 ± 2% (Figs 4 and 5,12 d). After 12 hours of rotation almost all of them became discocytes and 10% remained the same. In the other bag after 1 hour rotation almost all cells were echinocytes. And after 12 hours of rotation almost all of them became discocytes. This fact approved by the reversibility of these form of RBC. In other 6 bags the level of discocytes was 20–40% (1 h) and 50–70% (12 h). This is in good agreement with the reversibility of echinocytes into discocytes. In other bags we observed a little an other persantage of cell shapes, but we obtained the same tendency of recovery of cells. Percentage of irreversible forms (teardrop cells, spheroechinocytes) remained at the same level after 12 hours of rotation.

On the 19th day of PRBC storage, after 1 hour of rotation, there were already five species of PRBC. The majority of them were echinocytes and spheroechinocytes (up to 64 ± 16%) and teardrop cells (lemon-like) were present 10 ± 2%. After rotation during 12 hours the distribution of the cell forms changed. 76% of PRBC acquired the form of teardrop (lemon-like cells) elongated to one end or with typical two sharp ends on the one side of the cell (keratocytes). Additional image of such cells in an enlarged format is shown in Fig. 4, this is highlighted with a blue circle. Such PRBCs we observed earlier^18^. At the same time, spheroehinocytes were less than 15%, and discocytes were practically absent (less than 3%).

On 26 days of PRBC storage after 12 hours of rotation, the majority of cells were converted to spheroechinocytes (67 ± 8)%.

**Figure 6:**
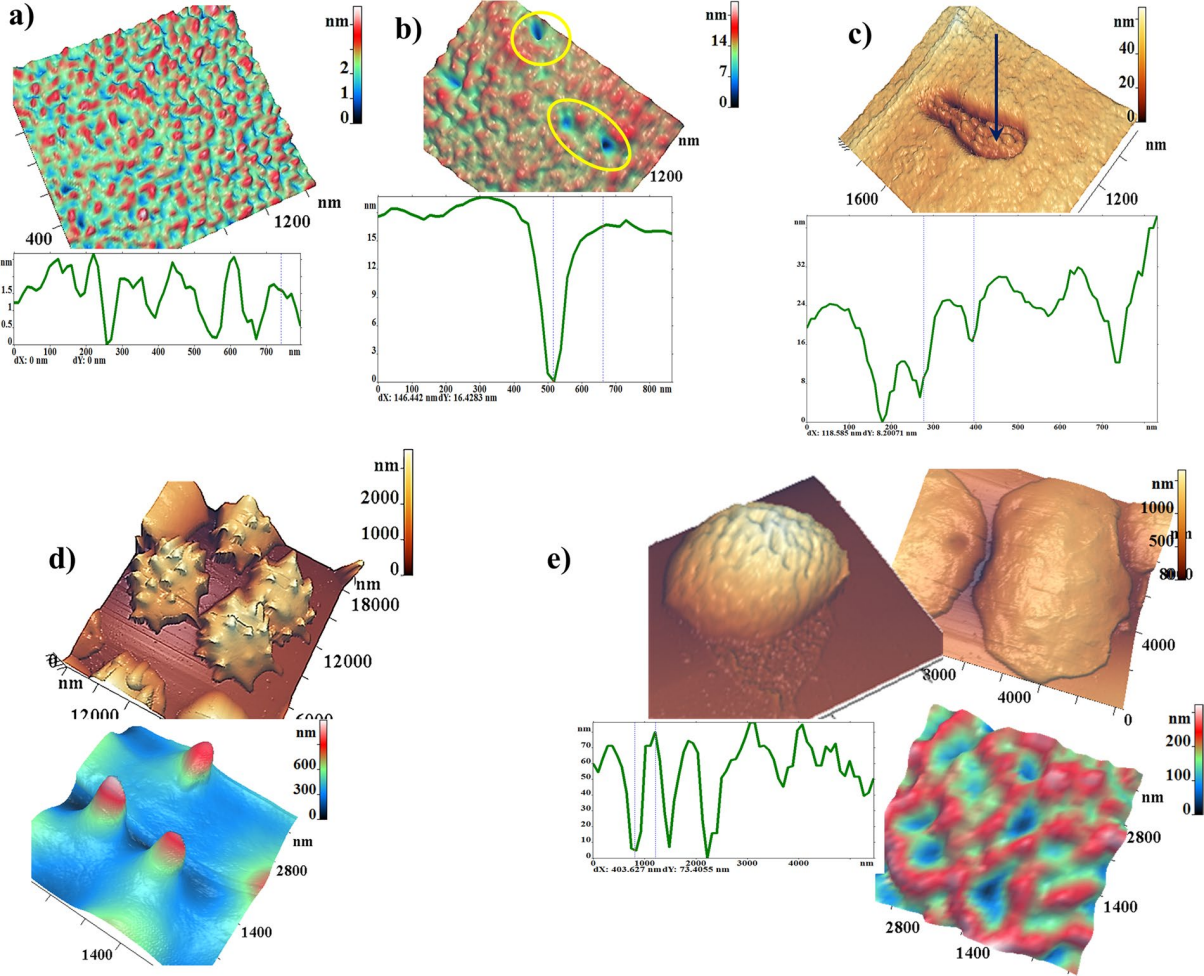
AFM-images of typical nanosurfaces of II order of PRBC membranes and corresponding nanosurface profiles. (**a**) For discocyte on the 5^th^ day of PRBC storage. (**b**) Single topological defects (highlighted by yellow circles) on the surface of membranes on the 16^th^ day of PRBC storage. (**c**) Domains with a grain-like structure.(**d**) Spheroehinocyte on the 33^th^ day of storage and its nanosurface. (**e**) Swelled cells e on the 40^th^ day of storage and its nanosurface.

And on day 40 there were 66% swelled and teardrop cells, 34% spheroechinocytes (Figs 5 and 6d,e).

Figure 5 shows the distribution of the main five forms of PRBC during 40 days of storage. In our experiments there were indicated: discocytes, echinocytes, spheroechinocytes, teardrop (dacrocytes) (lemon-like), swelled cells. Into discocytes group we included also stomatocytes, because their quantity was small, they are reversible and their elastic properties are approximately the same. Into dacrocytes group we included also cells like keratocytes. In each individual bag, the distribution of PRBC forms could differ from presented, but overall tendencies persisted.

- In this section we showed, that there is a transition period during long-term storage of PRBC, when morphology changes significantly and irreversible forms of PRBC appear. By time, it is nearly the transition period of the Young’s modulus.

### Nanostructure of membranes of PRBC

To obtain the characteristics of nanostructure of PRBC membranes fragments 0.5–1.5 μm^2^ with the highest possible AFM resolution of 1024 × 1024 points were scanned on the surface of a single cell. This surface was represented as the surfaces of I, II, and/or III orders of spatial Fourier transform^26^. In Fig. 6 there are shown AFM 3D-images of typical membrane nanosurfaces of discocyte (a), of membranes with topological defects (b,c) on 16 days of storage, of spheroechinocyte (d) and swelled cells (e) with corresponding membrane nanostructures. There are shown corresponding profiles of these surfaces.

The PRBC nanosurface in the initial storage period had no expressed topological defects, and the height of its roughness did not exceed 3 nm (Fig. 6a). Then, topological defects begin to appear on the surface of cells membranes, which are shown on Fig. 6b,c. Such defects appeared on the 16^th^–21^st^ day of PRBC storage for cells from the samples of all bags. The first type of topological defects are single dips with a depth of 12–20 nm with a outfall diameter 250–400 nm (Fig. 6b). On the visible surface of membranes such defects could appear in an amount from several units to several tens. The second type of topological defects are the domains with an internal grain-like structure (Fig. 6c). The domain sizes were varied in a wide range: from 400 to 2000 nm. The size of domain is determined by quantity of grains in it. The size of the grains is near 150 nm. The entire structure of the domain is lowered into the cell at 20–40 nm. Dimensions of topological defects and their details are presented on the corresponding surface profiles. Early we registered such domain structures under the action of toxins on RBC membranes^30^. In the second half of the period of PRBC storage topological defects were expanded and merged.

On the 26^th^–33^rd^ day of PRBC storage most cells in a monolayer were represented by spheroehinocytes. The membranes of these cells were large spicules with a height 500–1200 nm and with a diameter of 1000–1200 nm (Fig. 6d). By the 40^th^ day PRBC were transformed and the majority of PRBCs (greater than 50%) became swelled cells. The spicules were fallen off, and the cell size increased to 12 μm. The height of nanosurface was from 30 to 10 nm.

- In this section we showed that during long-term storage of PRBC there is a transition period when irreversible topological membrane nanodefects appear. The origin of these defects is the starting mechanism for the development of irreversible forms of PRBC. This precedes the transition period of the growth of Young’s modulus and the appearance of irreversible cell forms.

### Metabolic indicators

The concentration of K^+^ ions in the external solution increased during the storage. Initially, it was 3 ± 1 mM, and by the end of the storage period it increased to 16 ± 3 mM after 1 hour of rotation. This is in good agreement with the literature data^31^. It was 26 ± 4 after 12 hours of rotation.

The pH value decreased from 7.0 ± 2 at the beginning of storage to 6.6 ± 1 by the end of the period. During this time, the concentration of lactate increased to 20 mM. The initial glucose content in the hemopreservative solution - 26 ± 2.5 mM and decreased to 16 ± 4 mM.

We measured hemolysis by measuring of Hb with the analyzer ADVIA 60 and then calculating by standard formula^32^. Before 20 days the level of hemolysis did not excced 0,2% after sampling from bags and 0.4% after 12 h rotation. To the 40 day it was 0.75 ± 0.15% along 7 bags and in one bag it was 1%. Such сell loss could not affect on percentage distribution of cells in suspension.

## Discussion

We obtaine the main results using AFM and AFS. Atomic force microscopy has advantages over optical microscopy. It makes it possible to obtain images of objects near 1 nm in width and 0.2 nm in height. While the resolution limit of an optical microscope is limited by a light wavelength about 400–600 nm. Of course, the visual method is electron microscopy. But the need to perform certain operations for preparing samples (for example ref. 33) limits the ability to obtain a large number of images, excludes the possibility of obtaining an image of 100 cells and then one or more exactly from this array. In addition, it is impossible to obtain an electron microscopy image and immediately to measure the Young’s modulus on the same cell.

AFM and AFS are used by researchers^34^. The deformation properties of RBC are measured, for example, by a microfluidic slit-flow ektacytometer^35, 36^, optical tweezers^37^. However, no other methods allow the simultaneous registration of morphology, nanostructure and stiffness of membrane.

Young’s modulus of RBC membrane is the charactristics of membrane stiffness. Membrane stiffness influence on deformability. RBC deformability can influence on blood flow in the microcirculation and makes an important contribution to the transfusion outcome in humans^38^. Also decreased deformability may result in decreased capacity to pass through the spleen^23^.

In our experiments, we checked the biomechanical parameters an hour after the refrigerator. The temperature was raised gradually from 4 °C to 37 °C. This was done for all the samples on the relevant days. The results were compared for the same experimental conditions. We believe that the study of biomechanical properties at different temperatures may be the subject of the separate study. We heated PRBC based on the recommendations of AABB (USA). Blood components should be warmed if clinically indicated for situation such as massive transfusion, if rapid infusion of blood or blood components is needed in patients experiencing shock or surgical or anesthetic manipulations that disrupt temperature regulation^39^.

Rotation during 12 hours at the same temperature allowed us to analyze whether the biomechanical parameters are still constant or change and in what side. It was interesting to conduct from a biophysical point of view for different storage times. Indeed, we got results: in the first half of storage, until 20–26 days, cells after 12 hours of rotation partially restored their shape and membranes even improved elastic properties. And in the second half of storage, rotation did not lead to a decrease in the stiffness of the membranes and cells did not return to discocytes or stomatocytes.

In experiments *in vitro* we showed that during long-term storage of PRBC there is a transition period - 20–26 days, when the Young’s modulus of membranes grows abruptly, irreversible topological nanodefects appear, irreversible forms of RBC develop. By date these processes coincide (a sharp increase of E, irreversible morphology) or precede them (the appearance of membrane defects).

Another important result, which we have shown, is that before the transition period PRBC restore stiffness (*E* decreases) and morphology (to discocytes and other reversible forms). After the transition period, these indicators did not restored to initial levels in our experiments.

We checked the possibility of recovery of cells properties by rotation in buffer. This may be done also in rejuvenation solution^40^. But for such solution PRBC must be washed before infusion to remove inosine, which may be toxic^39^, which will cause additional artifacts of cell parameters.

In PRBC storage lesions have been linked to the oxidative stress. Cascade of events from metabolism to morphological changes arises and develops in RBC and solution due to oxidative processes^4, 9, 10^. Molecular mechanisms for appearance of the considered effects just in the transition period are of particular interest. Topological membrane defects that resulted from the destruction of cellular matrix were the precursors of the transition period^18, 30^. Exactly they become the cause of appearance of irreversible forms of RBC. At 19 day after 12 HR more than 75% of the cells in one bag were transformed into teardrop cells – cells with elongated sharpened ends (lemon-like cells). Some of them were as keratocytes. Teadrop cells are appeared due to oxidative process, caused by ultraviolet radiation for example^41^. In other bags the percentage of such cells were 40–50%. These phenomenon was typical for 12 hours of rotation. But for one bag teardrop cells were 40% after 1 hour of rotation. These facts show that these forms of cells are typical for this time of storage. But in different bags the process of shape change proceeds with different kinetics.

The appearance of such forms is probably associated with the processes of aggregation and crystallization of hemoglobin inside cells^42^. The growth of *E* could be caused by the appearance of membrane *Hb*_*m*_ and clustering of band 3^43, 44^. In addition, band 3 clustering causes the influx of IgG to cell, resulting in the reaction of immune system to RBC membranes as foreign objects^22, 45–47^. Namely these RBCs are primarily removed from the bloodstream under transfusion. Also the changed cells can be eliminated by the spleen^48^.

These phenomena are mainly caused by the development of oxidative processes in PRBC. After 19–21 days, the level of active glutathione decreased and the level of oxidized glutathione increased^10, 13, 18, 20, 49^, pH decreased to 6.7–6.45^17, 46, 50^. According to a number of authors, by the 21^st^ day, the level of 2,3-diphosphoglycerate reached almost zero^21, 46^, at the same time the concentration of malonic dialdehyde was almost doubled, that indicated on the processes of peroxidation of membrane lipids^24, 25^, the formation of vesiculation^46, 51–53^ and irreversible damages of membrane structures^54^. Oxidation processes led to disturbances in the spectrin matrix^18^. And cytoskeletal defects essentially influence on RBC shape^55–57^. The accumulation of Hb associated with the internal side of membrane^52^, that were harshly intensified in the second half of the storage period. As a consequence, topological nanodefects and irreversible morphological disturbances were appeared^18, 58, 59^.

Our results can be useful in assessing the quality of PRBC. The quality assessment of PRBC can be defined in the following way.

1. The value of Young’s modulus E must not exceed the control value (measured on the 2^d^–5^th^ day) 1.6–2.0 times;
2. In the monolayer of RBC the quantity of irreversible cells (spheroechinocytes, teardrop cells, swelled cells and other) must not exceed 25%. This corresponds to the criterion for the reduction of no more than 25% of cells *in vivo*. This corresponds to the basic criterion^8^.
3. Topological nanodefects are not registered on membranes surface.

The question is arisen:

Is the transition period necessarily occurs after 19^th^ day? Obviously no, may be earlier, may be later. The time of its occurrence depends on the initial quality of PRBC, the way of its donation and storage, the used hemopreservative solution, the impermeability of the blood bags, donor factors^60–62^ and others.

The proposed quality assessment is not technically complicated and by time and efforts it is comparable to a complex of biochemical and complete blood count test.

## Conclusion

The results presented in this work can be used in clinical medicine as additional characteristics for assessing of PRBC quality during its long-term storage. These presented data for quality assessment of PRBC can be effective for reducing of post-transfusion complications. This is especially important for PRBC transfusion to patients in critical state and with massive blood loss, for high-risk patients^63^. In our opinion, the study of the сomplex of phenomena in the transition period is of great scientific and practical interest and requires the conducting of separate additional studies. These data are also of interest for fundamental studies of molecular mechanisms of functioning and preservation of blood cells.

## Methods

### Packed red blood cells

PRBC in hermetic blood bags with SAGM preservative solution were obtained from four independent blood transfusion centers of Moscow Health Department. Donor whole blood was collected from donors on a voluntary basis in accordance with relevant guidelines and regulations of Russian Federation, informed consent was obtained from each donor. All experiments were carried out in accordance with guidelines and regulations of Federal Research and Clinical Center of Intensive Care Medicine and Rehabilitology, V.A. Negovsky Scientific Research Institute of General Reanimatology, Moscow, Russian Federation. All experimental protocols were approved by this Institute.

Totally 8 bags with RBC of blood groups O (I), A (II), B (III), AB (IV) (two containers of each blood group) were used in the study. PRBC was formed in accordance with existing instructions and standards: testing for infectious diseases, leukocyte reduction, hermetically packing into containers with a hemopreservative. Whole blood (450 ml ± 10%) was collected from healthy donors into CPD anticoagulant (63 mL) and leukodepleted. After separation of plasma by centrifugation, RBCs were suspended in 100 mL of SAGM (Saline, Adenine, Glucose, Mannitol) additive solution.

Hematocrit of PRBC was 60–65%.

PRBC were stored during 40 days at 4 °C in accordance with WHO recommendations^6^.

### General scheme of experiments

Measurements were made on “control” (2, 5, 12, 19, 26, 33 and 40), as well as some “intermediate” (16, 21) days of storage according to the scheme shown in Fig. 1. Samples of PRBC were taken from each blood bag without damaging its hermeticity and diluted with a solution of phosphate buffer pH = 7.4 (MP Biomedicals LLC, France) to normal hematocrit (40–46%). The amount of volume is important. Directly for measurements of morphology, nanostructure and stiffness, 100 μl is sufficient. But for rotation of the suspension we used 10 ml. In days when biochemical indicators were measured 24 ml were taken.

Tubes with samples of PRBC were placed on rotator “Bio RS-24” (Latvia) with rotation frequency 6–8 rpm, placed into the thermostat at 37 °C. After reaching the set temperature of PRBC (1 hour), 0.5 of volume was taken from each tube for carrying out measurements. These fragments are indicated in Fig. 1 by the index 01HR - 1 hour of rotation. The remaining volume of PRBC was also stored in the thermostat on the rotator for 12 hours. These fragments are indicated in Fig. 1 by the index 12HR - 12 hours of rotation.

The samples 01HR and 12HR were used to create dry RBC monolayers (DS Fig. 1) for obtaining AFM images, to create monolayers of native cells in a liquid medium (LS Fig. 1) for measuring of stiffness of membranes and to obtain samples for analysis (SFA Fig. 1). Such scheme of experiments was carried out for each of the 8 bags, for the temperature of 37 °C, for each control storage days. According to this scheme, during the entire experiment, 56 experiments were conducted for control days and 12 for additional days.

### Obtaining of monolayers

Monolayers of RBC were formed for studying the nanostructure of membrane surface by the method of atomic force microscopy AFM (DS Fig. 1) and for measurements of the Young’s modulus of membranes by atomic force spectroscopy (LS Fig. 1). Monolayers for subsequent processing can be obtained either by smearing on a glass (V-Sampler Vision, AU), or by using special centrifuges (Statspin Diffspin-2, USA). However, these methods damage the cell and deform the membranes. In our work we used sedimentation method to obtain RBC monolayers. This method, firstly, is not traumatic with respect to cells and their membranes, and, secondly, it allows to prepare simultaneously monolayers of both native and dry RBC.

50 μl of PRBC were added into 5 ml of phosphate buffer and an erythrocyte suspension was created. 250 μl of suspension was applied on the glass with polylysine, held for 10 minutes and then washed in buffer. After that, the precipitate was fixed in 0.3% glutaraldehyde, washed again in buffer. Part of these samples were placed into AFM cuvette with a buffer (LS) to measure the Young’s modulus of native cell membranes. The other part was dried at room temperature in air (DS) and was used to obtain images of membranes nanostructure and cells.

### Atomic force microscopy and spectroscopy

The images of the cells and nanostructure of their membranes (DS Fig. 1) were obtained using an atomic force microscope “NTEGRA Prima”, (NT-MDT, Russian Federation) in a semicontact mode. The cantilevers NSG01 (Nanosensors, Switzerland) with force constant 5 N/m and tip radius 10 nm were used. The number of scan points is 1024 with in each line of image. Scanning fields: 120 × 120 μm^2^, 10 × 10 μm^2^, 3 × 3 μm^2^. In some cases, scanning fields with the size up to 500 × 500 nm were made to obtain high-quality images of nanostructure of the membranes. Images and their profiles were analysed in 2D- and 3D-formats. For the analysis of nanostructure of RBC membranes, there were used nanosurfaces of I, II and III orders by spatial Fourier transform^26^.

The typical membrane parameters of each image were quantified by the software FemtoScan Online (Advanced Technologies Center, Moscow, Russian Federation).

Stiffness of the cells was measured using atomic force spectroscopy method^27^. For this purpose cantilevers SD-R150-T3L (Nanosensors, Switzerland) with force constant 0.15 N/m and tip radius 150 nm were used. In measurements of Young’s modulus of cell membranes, cantilevers with a large radius of the probe were used so as not to disturb the membrane structure (a probe with a radius of 10 nm punctures the membranes). The measured magnitude of the absolute value of *E* depends on the properties of the RBC themselves and on many external experimental factors: the method of preparation of monolayers, the fixation time, the method of washing of samples, the conditions of holding in the liquid, the temperature, the immersion rate of the probe, and a number of other parameters. Its value differs in the works of various authors in tens, if not hundreds, times^27, 64^. Therefore, for the possibility of comparing the results, all measurements were carried out strictly in the same experimental conditions: all measurements were carried out at a depth of probe immersion of 50 nm, during 5 s of probe action on the membrane.

The erythrocyte membrane is an elastic deformable material^27, 45^. The empirical force curve obtained by AFM is the dependence of photodiode deflection current *I* on the magnitude of the vertical displacement Z of the piezo-scanner. The transition to the function F (Z) was carried out according to the expression:

1 $$F(Z)=\frac{{I}_{m}}{{I}_{g}}K\,Z,$$

where: *F* is the force acting on the membrane, *K* is the cantilever force constant (N/m), *I*_*m*_ and *I*_*g*_ are the deflection currents when the indenter is acting on the membrane and glass (stiff material), respectively. The Young’s modulus of the local region of the membrane was calculated using the Hertz formula:

2 $$F=4/3\,E\,{R}^{1/2}{h}^{3/2}$$

where: *E* is the Young’s modulus (N/m^2^), R is the radius of the probe, and *h* is the immersion depth of the probe.

### SFA analysis methods

The complete blood count test analysis was carried out on the analyzer «ADVIA 60» (Germany), biochemical analysis - on the analyzer «Miura One» (I.S.E. Group, Italy). Analysis of acid-base state was carried out with help of an ionometric converter “I-510” (RF). The electrodes ES-10603 with the reference electrode ESr-10103 were used for pH measurement. The concentration of potassium ions was measured by the potassium-selective electrode XC-K-001.

### Statistical analysis

#### Cells morphology

5 scans of 100 × 100 μm^2^ were received on each sample of each PRBC bag (DS_1_ and DS_12_). The morphology for 70–90 cells (on an average 800 cells for DS_1_ and DS_12_) was analyzed on each scan. There were considered 5 main forms of RBC for which statistical analysis was performed – dicocytes, echinocytes, spheroechinocytes, o teardrop (lemon-like) cells, swelled cells. For 7 control days of measurements the total statistical ensemble was more than 6000 cells for one bag. The whole ensemble of RBC was 48000.

#### Measurement of Young’s modulus

2 scans of 100 × 100 μm^2^ of native RBC in a liquid medium were received on each sample of each RBC bag (LS_1_ and LS_12_). On each scan stiffness was measured on 60 cells (an average of 120 cells for one sample). For the temperature of 37 °C (1HR, 12HR) more than 240 cells were analyzed. For 7 control days of measurements the total statistical ensemble was more than 1680 cells for one blood bag. The entire ensemble of Young’s modulus measurements was 13450 RBC.

For each control storage day and rotation regime the obtained data were averaged over 8 bags. Average Young’s modulus (taking into account equations 1 and 2), standard deviation were measured, histograms of the density of relative frequency were plotted. Approximation of the histograms was carried out by the normal law of Gaussian distribution. The one-way ANOVA was used, *p < 0.05 was considered statistically significant. The data were statistically processed using program Origin (OriginLab, Northampton, MA).

#### SFA analyzes conducting

pH, concentration of extracellular K^+^ were measured for each bag. The obtained data was averaged over 8 bags for each day of measurement.
